# Initial Experience of Vericiguat Treatment in Patients with Heart Failure and Reduced Ejection Fraction

**DOI:** 10.3390/jcm12134396

**Published:** 2023-06-29

**Authors:** Makiko Nakamura, Teruhiko Imamura, Koichiro Kinugawa

**Affiliations:** Second Department of Internal Medicine, University of Toyama, Toyama 930-0194, Japan; nakamura@med.u-toyama.ac.jp (M.N.); kinugawa@med.u-toyama.ac.jp (K.K.)

**Keywords:** congestive heart failure, HFrEF, SGLT2 inhibitor, sacubitril/valsartan

## Abstract

Background: Vericiguat, a novel oral soluble guanylate cyclase stimulator, decreased the incidence of cardiovascular death and heart failure hospitalization in the cohort receiving triple therapy for heart failure with reduced ejection fraction. However, efficacy and optimal patient selection in real-world practice in the era of “quadruple therapy” remains unknown. Methods: Consecutive patients who received vericiguat between September 2021 and December 2022 were retrospectively evaluated. Results: A total of 28 patients (median age 66 years, median left ventricular ejection fraction 33%) were included. Of them, 21 patients (75%) received quadruple therapy, and the dose of sacubitril/valsartan was 100 mg in the median. Three patients receiving dobutamine infusion discontinued vericiguat due to symptomatic hypotension. One patient with a high N-terminal pro-B-type natriuretic peptide of 4848 pg/mL at the baseline underwent unexpected heart failure hospitalization. Efficacy was evaluated in 21 patients who continued vericiguat for more than 4 months. The plasma B-type natriuretic peptide concentration tended to increase during the six-month pre-treatment period from 104 pg/mL to 179 pg/mL on median (*p* = 0.665) but remained unchanged after six-month vericiguat treatment to 170 pg/mL on median (*p* = 0.188). Conclusions: Vericiguat therapy might be feasible and effective upon quadruple therapy for those with heart failure, although further investigation is warranted to validate our findings.

## 1. Introduction

Vericiguat, a novel oral soluble guanylate stimulator, enhances the cyclic guanosine monophosphate (GMP) pathway by directly stimulating soluble guanylate cyclase independent of nitric oxide. In addition, vericiguat sensitizes soluble guanylate cyclase to endogenous nitric oxide by stabilizing the nitric oxide binding site [1,2].

A reduction in death from cardiovascular causes and heart failure hospitalization by vericiguat therapy over placebo was demonstrated in patients with heart failure with reduced ejection fraction (HFrEF) accompanying a high risk of worsening heart failure in the VICTORIA trial [2,3]. In this trial, 59.7% of them received triple therapy consisting of beta-blockers, renin-angiotensin system inhibitors, and mineralocorticoid receptor antagonists. The number of patients who received sacubitril/valsartan was small (14.5%), and the dose of sacubitril/valsartan was not reported. Patients with systolic blood pressure below 100 mmHg or those receiving intravenous catecholamine infusion were excluded [2,3].

The DAPA-HF and EMPEROR-Reduced trials demonstrated that sodium-glucose cotransporter-2 (SGLT2) inhibitors reduced the combined risk of cardiovascular death and heart failure hospitalization in patients with HFrEF, irrespective of the existence of diabetes [4,5,6].

The number of patients receiving SGLT2 inhibitors in the VICTORIA trial was relatively small [3], and the efficacy and safety of vericiguat therapy in patients receiving “quadruple therapy” including SGLT2 inhibitors [7], a gold standard medical strategy for patients with HFrEF in the current era, remained uncertain. In this study, we reported the safety and efficacy of vericiguat therapy, which was added to quadruple therapy in most cases in real-world practice, and discussed the optimal patient selection for vericiguat therapy.

## 2. Methods

### 2.1. Patient Selection

**Figure 1:**
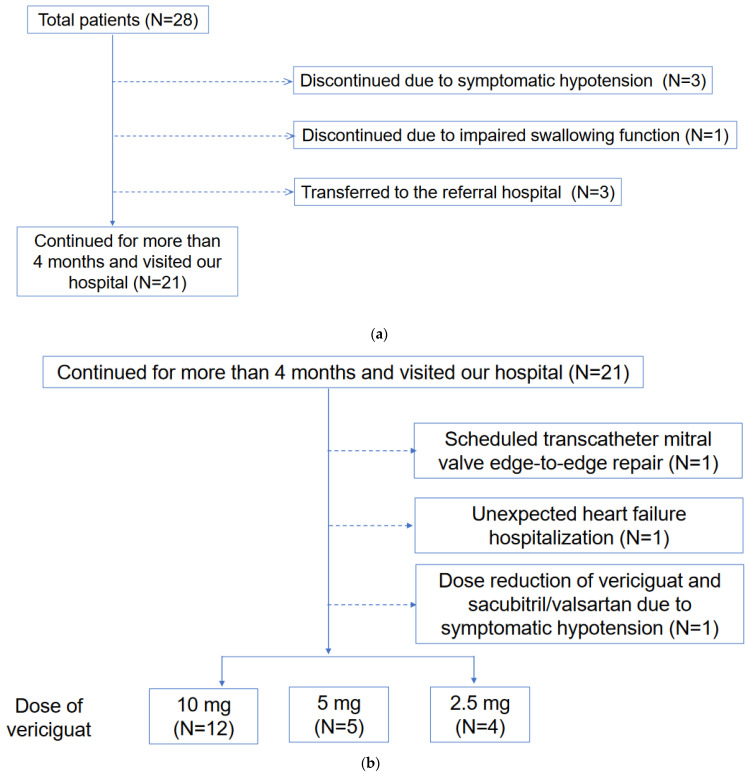
(**a**) Patient selection for efficacy assessment is shown. (**b**) Clinical course including adverse events among patients included efficacy assessment shown.

This retrospective study included patients who received vericiguat between September 2021 and December 2022. Safety analysis was performed on all patients. Efficacy analysis was performed for those who continued to receive vericiguat for more than 4 months and visited our hospital (Figure 1). This study was conducted in accordance with the “Declaration of Helsinki” and was previously approved by the local institutional review board (IRB number R2015154). Written informed consent was wavered due to the retrospective nature of this study and the opt-out of this study. 

### 2.2. Vericiguat Therapy

Patients with HFrEF who presented with symptoms of worsening heart failure against guideline-directed medical therapy were eligible to receive vericiguat in the outpatient clinic or during the index hospitalization at the discretion of the attending cardiologists. 

The dose titration of vericiguat was performed according to the manufacturer-recommended regimen. Vericiguat started at 2.5 mg and was up-titrated to 10 mg. If patients had systolic blood pressure below 90 mmHg or hypotensive symptoms, including dizziness and lightheadedness, the dose up-titration was postponed. 

### 2.3. Collected Data

Baseline characteristics, including laboratory, echocardiographic, and medication data were collected. Several laboratory data, were also obtained 6 months before, and 6 (±2) months after the initiation of vericiguat. 

### 2.4. Statistical Assessments

Statistics were performed using JMP pro ver17.0 (SAS Institute Inc). Variables with *p* < 0.05 were considered significant. Continuous data were described as the median and interquartile ranges and were compared between two groups using the Mann–Whitney U-test. Categorical data were compared between the two groups by Chi-square test or Fischer’s exact test as appropriate. In 21 patients who continued vericiguat treatment for over 4 months, clinical data were compared between the baseline, 6 months before and 6 (±2) months following vericiguat therapy using a Wilcoxon singed-rank test. 

## 3. Results

### 3.1. Baseline Characteristics (Safety Analysis)

All 28 patients who received vericiguat were assigned to the safety cohort. The median age was 66 (56, 79) years old, and 23 were men (Table 1 and Table 2). In total, 27 patients (96%) received renin-angiotensin system inhibitors, including 23 receiving sacubitril/valsartan. In total, 27 patients (96%) received beta-blockers, and 25 patients (89%) received mineralocorticoid receptor antagonists. In total, 22 patients (79%) received SGLT2 inhibitors. Among the 6 patients who had not received SGLT2 inhibitors at the time of administration of vericiguat, 2 patients had discontinued SGLT2 inhibitors due to refractory diarrhea and inhibited oral intake for unstable hemodynamics, respectively, and the other 4 patients did not receive any due to frailty. In total, 18 patients (85%) received tolvaptan due to the treatment of fluid retention, which did not respond well to loop diuretics and/or accompanying hyponatremia [8].

### 3.2. Safety Analysis

#### 3.2.1. Termination of Vericiguat and Dose Titration

Three patients who initiated vericiguat while receiving continuous dobutamine infusion terminated vericiguat due to symptomatic hypotension. Another elderly patient with an impaired swallowing function refused to continue vericiguat and discontinued it. The other 3 patients were transferred to the referral hospital (Figure 1a).

Of all the patients, 21 continued vericiguat for over 4 months. A patient had a dose reduction from 10 mg to 5 mg due to symptomatic hypotension. One patient reduced the dose of sacubitril/valsartan from 200 mg to 100 mg due to symptomatic hypotension. As of the study’s end, 12 patients (57%) had 10 mg, 5 patients (24%) had 5 mg, and 4 patients (19%) had 2.5 mg of vericiguat (Figure 1b). 

#### 3.2.2. Any Interventions

There was no cardiovascular death. One patient with plasma B-type natriuretic peptide of 239 pg/mL and serum N-terminal pro-B-type natriuretic peptide of 4848 pg/mL and mildly impaired renal function with an estimated glomerular filtration ratio of 47.7 mL/min/1.73 m^2^ at the baseline had unexpected heart failure hospitalization after 42 days following vericiguat administration (Figure 1b). The patient received triple therapy, consisting of enalapril, bisoprolol, and spironolactone, whereas sacubitril/valsartan and the SGLT2 inhibitor were not administrated. After hospitalization, the patient was initiated with 50 mg of sacubitril/valsartan and an SGLT2 inhibitor in addition to 2.5 mg of vericiguat. There was no further heart failure rehospitalization. 

One patient underwent scheduled transcatheter mitral valve edge-to-edge repair as an additional non-pharmacological treatment for functional severe mitral regurgitation 4 months after the administration of vericiguat therapy (Figure 1b). One patient was up-titrated with a beta-blocker from a 20 mg to 35 mg carvedilol equivalent. 

#### 3.2.3. Baseline Characteristics (Efficacy Analysis)

A total of 21 patients who continued vericiguat for over 4 months were included in the efficacy analysis. The baseline characteristics are summarized in Table 1 and Table 2. The median age was 67 (59, 79) years, and 17 were men. The median heart failure duration was 5 (2, 10) years, and 16 patients (76%) had a history of heart failure hospitalization within a year. The median left ventricular ejection fraction was 34% (26%, 38%), and plasma B-type natriuretic peptide was 179 (118, 406) pg/mL. 

#### 3.2.4. Efficacy Analysis

**Figure 2:**
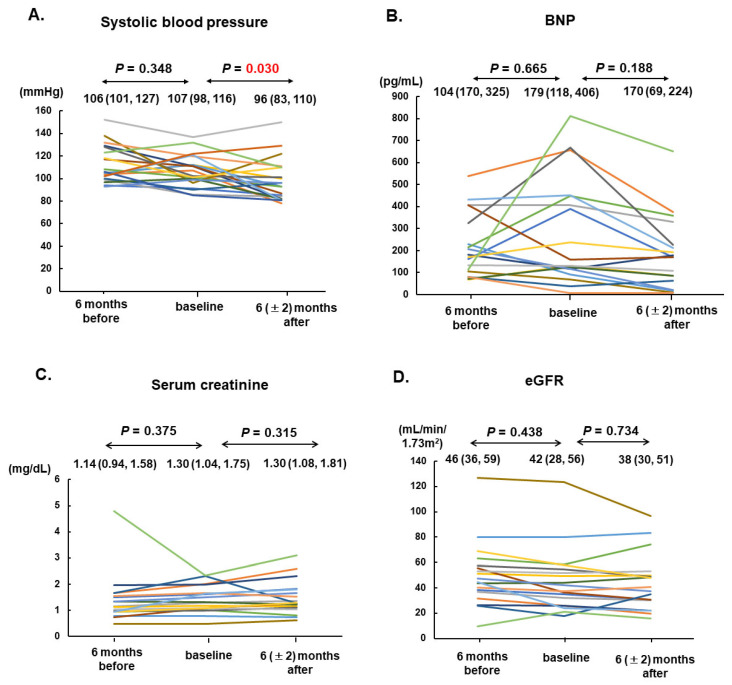
Trends of systolic blood pressure (**A**), serum BNP (**B**), serum creatinine (**C**), and eGFR (**D**) among 21 patients at 6 months before, at the baseline, and 6 (±2) months after vericiguat therapy are shown. The individual transition of each values were shown by different colors. BNP, B-type natriuretic peptide; eGFR, estimated glomerular filtration rate.

Vericiguat was administered for 236 days as the median. The maintenance dose was 10 (5, 10) mg. In total, 19 patients (90%) received sacubitril/valsartan at 100 mg as the median. All patients received beta-blockers at a 10 mg of carvedilol equivalent dose as the median and mineralocorticoid receptor antagonists. In total, 17 patients (81%) received SGLT2 inhibitors. Systolic blood pressure declined significantly from 107 (98, 116) mmHg down to 96 (83, 119) mmHg (*p* = 0.030; Figure 2A). 

The plasma B-type natriuretic peptide concentration tended to increase during the six-month pre-treatment period from 104 pg/mL to 179 pg/mL on median (*p* = 0.665); however, they remained unchanged after six months of vericiguat treatment from 179 pg/mL to 170 pg/mL on median (*p* = 0.188; Figure 2B).

Serum creatinine and the estimated glomerular filtration rate remained unchanged during the pre-treatment period and during vericiguat treatment (*p* = 0.314 and 0.734, respectively; Figure 2C,D). 

## 4. Discussion

In this study, we investigated the safety and efficacy of vericiguat in patients with HFrEF in real-world practice. Vericiguat was up-titrated to a targeted dose in around half of the patients and was terminated in a few patients due to hypotension. Plasma B-type natriuretic peptide remained unchanged following 6 months of vericiguat therapy, although it tended to increase in the pre-treatment period.

### 4.1. Patient Selection on the Viewpoint of Feasibility

Given the mechanism of vericiguat, which dilated the systemic vasculature, it was plausible that vericiguat lowered blood pressure. The VICTORIA trial did not include patients with systolic blood pressure below 100 mmHg and those receiving intravenous inotropes [3], both of which were included in our study to clarify real-world data. Based on our findings, patients receiving intravenous inotropes might not be good candidates for vericiguat therapy. 

The PARADIGM-HF trial demonstrated the survival benefit of sacubitril/valsartan over enalapril in patients with HFrEF [9], and any dose reduction was associated with a higher subsequent risk of cardiovascular death and heart failure hospitalization [10]. In our study, the median dose of sacubitril/valsartan was 100 mg due to a relatively lower baseline systolic blood pressure. Patients who were intolerant to the up-titration of sacubitril/valsartan to 400 mg but who had an incremental natriuretic peptide concentration might be good candidates for vericiguat for further risk reduction.

In the VICTORIA trial, renal function trajectories were reported to be similar between vericiguat- and placebo-treated patients, and the beneficial effects of vericiguat on the primary composite outcome of cardiovascular death or heart failure hospitalization were consistent across the full range of the estimated glomerular filtration rate [11]. In our study, the baseline estimated glomerular filtration rate was reduced to 42 mL/min/1.73 m^2^ and remained unchanged during the vericiguat treatment, similar to the VICTRIA trial, while our cohort included patients with a baseline systolic blood pressure below 100 mmHg.

Vericiguat therapy might be feasible even in patients with a baseline systolic blood pressure below 100 mmHg if not receiving intravenous inotropes, and careful attention was paid to reactive hypotension.

### 4.2. Dose Adjustment in Real-World Practice

The SOCLATES-REDUCED phase II randomized trial demonstrated a dose-dependent improvement in N-terminal pro-B-type natriuretic peptide levels and a left ventricular ejection fraction up to 10 mg of vericiguat [12], and 10 mg is commercially recommended as a target dose of vericiguat. However, in this study, only 60% of the participants received triple therapy. No patients received SGLT2 inhibitors to treat their heart failure. If many patients received vericiguat upon completing quadruple therapy [7], it was questionable whether many patients could achieve the target dose of 10 mg vericiguat. Consistently, only 57% of our patients achieved the target dose of 10 mg due to hypotension. Special attention should be paid to the up-titration of vericiguat up to the maximum dose, particularly in patients with triple or quadrat therapy. 

### 4.3. Patient Selection on the Viewpoint of Efficacy

The readmission rates within one year of discharge for patients hospitalized with heart failure were reported as high as 25% previously [13]. Our cohort had a higher rate of prior heart failure hospitalizations within a year; however, there was only one patient (5%) who had an unexpected readmission during the observational period. This patient had an extremely high baseline serum N-terminal pro-B-type natriuretic peptide level. In a prespecified analysis of the VICTORIA trial, the patient with the highest quartile of the N-terminal pro-B-type natriuretic peptide (>5314 pg/mL) had fewer benefits from vericiguat therapy [3]. Although additional analysis reported that a reduction in the primary composite endpoint and its cardiovascular death and heart failure hospitalization components was observed in patients on vericiguat compared with subjects on a placebo with N-terminal pro-B-type natriuretic peptide levels up to 8000 pg/mL [14], patients with a higher N-terminal pro-B-type natriuretic peptide over 4000 pg/mL, might be better when initiating SGLT2 inhibitor beforehand. 

In our cohort, most of the patients received quadruple therapy, and the baseline N-terminal pro-B-type natriuretic peptide was relatively lower when compared to the VICTORIA trial. There might be some clinical benefit of vericiguat for the reduction in the residual risk after quadruple therapy; however, patients receiving intravenous inotropes with New York Heart Association class IV discontinued vericiguat and were not included in the efficacy analysis. Therefore, the clinical benefit of vericiguat in more severely symptomatic or sicker patients may be limited. We cannot derive any conclusions given a short observational period for a small sample-sized cohort. Major recipients have advanced heart failure, and the impact of vericiguat on cost-effectiveness, quality of life, and exercise capacity remains the next concern.

### 4.4. Study Limitations

This study was conducted retrospectively in a very small sample size cohort at a single institute, and the observation period was just 6 months. Some differences in clinical implications might not reach statistical significance due to a small sample size. We lacked a control group, given that it was not ethically allowed when treating patients with advanced heart failure without vericiguat in the current era. We, in this study, assessed the clinical implication of vericiguat-incorporated medical therapy. We could not exclude the impact of other medications.

## 5. Conclusions

Vericiguat might be feasible and effective in patients with HFrEF when receiving quadruple therapy without receiving intravenous inotropes and having difficulty in up-titration in first-line agents, including sacubitril/valsartan, while careful attention should be paid to hypotension. Further studies are warranted to validate our hypothesis.

## Figures and Tables

**Table 1:** Baseline demographic data.

	Safety Analysis (N = 28)	Efficacy Analysis (N = 21)
Age (years)	66 (56, 79)	67 (59, 79)
Male sex	23 (82%)	17 (81%)
Time from diagnosis of heart failure (years)	4.8 (1.4, 10)	5 (2, 10)
Heart failure hospitalization within a year	14 (50%)	16 (76%)
Body mass index (kg/m^2^)	24.1 (21.0, 26.8)	23.0 (20.7, 25.9)
New York Heart Association classification		
class II	15 (54%)	13 (62%)
class III	9 (32%)	8 (38%)
class IV	4 (14%)	0 (0%)
Dilated cardiomyopathy	10 (36%)	8 (38%)
Hypertrophic cardiomyopathy, dilated phase	5 (18%)	5 (24%)
Ischemic etiology	3 (11%)	3 (14%)
Cardiac sarcoidosis	2 (7%)	2 (10%)
Atrial fibrillation	4 (14%)	3 (14%)
Diabetes mellitus	3 (11%)	1 (5%)
ICD/CRTD	12 (43%)	9 (43%)
Percutaneous mitral valve repair	3 (11%)	3 (14%)
Systolic blood pressure (mmHg)	102 (95, 114)	107 (99, 102)
Diastolic blood pressure (mmHg)	67 (58, 74)	66 (57, 74)
Heart rate (bpm)	73 (70, 80)	70 (69, 80)

ICD, implantable cardioverter-defibrillator; CRTD, cardiac resynchronization therapy with defibrillation. bpm, beats per min. Continuous variables are presented as median and interquartile ranges. Categorical variables are presented as the number and percentage.

**Table 2:** Baseline laboratory data, echocardiographic data, and medications.

	Safety Analysis (N = 28)	Efficacy Analysis (N = 21)
Laboratory data		
Blood urea nitrogen (mg/dL)	21.2 (16.9, 30.6)	21.2 (17.8, 31.9)
Serum creatinine (mg/dL)	1.31 (1.06, 1.67)	1.30 (1.07, 1.65)
Estimated glomerular filtration rate (mL/min/1.73 m^2^)	43.4 (30.4, 58.2)	42.1 (29.7, 54.8)
Plasma B-type natriuretic peptide (pg/mL)	190 (124, 416)	179 (118, 406)
Serum N-terminal pro B-type natriuretic peptide (pg/mL)	903 (583, 2437)	920 (587, 1970)
Hemoglobin (g/dL)	13.6 (12.5, 14.2)	13.6 (12.5, 14.2)
Echocardiographic data		
Left ventricular end-diastolic diameter (mm)	64 (56, 67)	64 (55, 66)
Left ventricular ejection fraction (%)	33 (24, 37)	34 (26, 38)
Medication		
ACEI or ARB	4 (14%)	2 (10%)
Sacubitril/valsartan	23 (82%)	19 (90%)
Dose of sacubitril/valsartan (mg)	100 (50, 100)	100 (50, 100)
Beta blocker	27 (96%)	21 (100%)
Dose of beta blocker (mg; carvedilol equivalent)	11.3 (9.4, 20)	10 (10, 20)
Mineralocorticoid receptor antagonist	25 (89%)	21 (100%)
SGLT2 inhibitor	22 (79%)	17 (81%)
Loop diuretics	18 (64%)	15 (71%)
Tolvaptan	18 (84%)	14 (67%)

ACEI, angiotensin converting inhibitor; ARB, angiotensin receptor blocker; SGLT2, sodium-glucose cotransporter-2. Continuous variables are presented as median and interquartile ranges. Categorical variables are presented as number and percentage.

## Data Availability

Data are available from the corresponding author upon reasonable request.
